# Resilience and post-traumatic stress symptoms in grandparents following their grandchild’s cancer diagnosis from a multicenter cohort study in Switzerland (The GROKids project)

**DOI:** 10.1007/s12672-026-04490-7

**Published:** 2026-02-01

**Authors:** Peter Francis Raguindin, Anne Maas, Anica Ilic, Cristina Priboi, Katharina Roser, Ahmed Farrag, Freimut Schilling, Ursula Tanriver, Tamara Diesch-Furlanetto, Katrin Scheinemann, Gisela Michel

**Affiliations:** 1 Faculty of Health Sciences and Medicine, University of Lucerne, Lucerne, Switzerland; 2 Faculty of Medicine, Institute of Basic Medical Sciences, University of Oslo, Oslo, Norway; 3 Veterinary Public Health Institute, University of Bern, Bern, Switzerland; 4 Division of Pediatric Hematology and Oncology, Department of Pediatrics, Children’s Hospital of Central Switzerland, Lucerne, Switzerland; 5 Pediatric Oncology Department, South Egypt Cancer Institute, Assiut University, Assiut, Egypt; 6 Department of Pediatric Oncology and Hematology, University Children’s Hospital Basel, Basel, Switzerland; 7 Division of Hematology-Oncology, Children’s Hospital of Eastern Switzerland, St Gallen, Switzerland

**Keywords:** Resilience, Post-traumatic stress, Grandparents, Family, Cancer, Childhood cancer

## Abstract

**Background:**

Resilience is the dynamic ability to adapt to adversity using personal and social resources. Childhood cancer represents a major family stressor, and grandparents often provide emotional, practical, and financial support. Yet, their psychosocial outcomes and resilience remain poorly understood. We aimed to: (1) identify resilience trajectories (2), examine their association with post-traumatic stress symptoms, and (3) determine factors influencing resilience.

**Methods:**

This multicenter cohort study included grandparents of children recently diagnosed with cancer and treated at one of eight participating pediatric oncology centers in Switzerland. Eligible grandparents were recruited and completed questionnaires at 3-, 6-, 12-, and 24- months post-diagnosis. Resilience (CD-RISC 10), post-traumatic stress symptoms (IES-R), information needs, health literacy (HLS-EU-Q12), partnership quality, and social support (MSPSS) were measured. We used group-based trajectory modeling to identify resilience trajectories (Aim 1), linear mixed models to examine associations of resilience trajectories with post-traumatic stress symptoms (Aim 2), and linear mixed-effects models to identify the internal and external resources for resilience (Aim 3).

**Results:**

We included data of 41 grandparents of 20 children with cancer. Mean age was 67.6 years; most were grandmothers (*n* = 25, 61%), unemployed or retired (*n* = 23, 59%), and partnered (*n* = 35, 90%). Two resilience trajectories emerged within two years after diagnosis: low-stable (*n* = 17, 43%) and high-declining (*n* = 23, 57%). Grandparents in the low-stable group reported significantly higher post-traumatic stress symptoms (β: -19.8, 90% CI -29.2, -10.4, *p* < 0.001). The following internal resources were positively associated with resilience: higher health literacy (β: 0.31, 90% CI 0.20, 0.42, *p* < 0.001), more information received (β: 1.53, 90% CI 1.27, 1.79, *p* < 0.001), and having income that meets needs (β: 7.56, 90% CI 1.86, 13.26, *p* = 0.029). No external resources showed significant associations.

**Conclusion:**

Timely, clear, and tailored information may help strengthen grandparents’ resilience and reduce stress.

**Supplementary Information:**

The online version contains supplementary material available at 10.1007/s12672-026-04490-7.

## Introduction

Resilience can be defined as “the process and outcome of successfully adapting to difficult or challenging life experiences, especially through mental, emotional, and behavioral flexibility and adjustment to external and internal demands.” [1] It reflects an ongoing process of adaptation, shaped by both internal and external resources, that enables individuals to maintain or regain functioning in the face of adversity [2, 3]. Resilience is not a fixed trait but a dynamic process that varies across life stages and contexts [2, 3]. Studies have shown that resilience increases with age [4], possibly due to accumulated life experiences that are shaped through time. Older adults rely on both internal (personal) and external (social) psychological resources that help them maintain stability during stress [5, 6]. High resilience in this age group is hypothesized to be linked to lower post-traumatic stress symptoms, largely through the use of effective coping strategies that support adaptive functioning after adversity [7, 8].

The diagnosis of childhood cancer is a stressful event for affected children and their families, particularly in young children aged 4 to 12 years [9–11]. Within the family system, grandparents are often involved in the care of their grandchildren from the time of diagnosis through the course of treatment [11, 12]. In Switzerland, for example, almost half of all grandparents provide regular childcare support at least once a week [13], suggesting a high level of involvement in family life. Given this substantial engagement, grandparents assume additional caregiving responsibilities when a child or another family member becomes ill [11]. Despite this, few studies have investigated the psychosocial impact of childhood cancer on grandparents [10].

Given the intensity of the psychosocial impact of childhood cancer diagnosis on the family, understanding how grandparents cope with these challenges is essential. A cancer diagnosis in a child can lead to a series of changes within the family, such as changes in childcare responsibilities, household chore responsibilities, employment and financial resources [14–16]. This may require family members, including grandparents, to adapt in various ways. While several internal (personal) factors, such as economic status, general health, health literacy, and information provision [17], as well as external (social) resources, such as partnership status and quality, religious affiliation, and perceived social support [18], had been shown to contribute to resilience, their role in grandparents of children with cancer is yet unknown.

To date, no studies have explored resilience in older adults whose grandchildren are diagnosed with a serious illness. In particular, no research has investigated resilience trajectories following a childhood cancer diagnosis, nor how these trajectories influence post-traumatic stress reactions in grandparents.

Therefore, we aimed to: 1) identify trajectories of resilience in grandparents of children with cancer, 2) determine the association between resilience trajectories and post-traumatic stress symptoms, and **3**) identify internal (personal) and external (social) resources and contextual factors that influence resilience.

## Methods

This was a multicenter study from a cohort of grandparents whose grandchildren were recently diagnosed with cancer in Switzerland [19]. We followed the STROBE Statement Checklist for transparent reporting of study methods and results [20].

### Sample and procedures

We included grandparents whose grandchild was recently diagnosed with cancer and was undergoing cancer treatment. Eligibility criteria included having a grandchild who: was under 18 years of age, had received a cancer diagnosis within the past three months, was treated at one of eight participating pediatric oncology centers in Switzerland, and the grandparent was fluent in German, French, or Italian.

Recruitment was facilitated through hospital staff at the participating clinics, who either directly approached eligible grandparents or asked parents to provide contact information for the grandparents. Upon contact with the study team, grandparents received an information letter detailing the study’s objectives along with a consent form. We sent paper questionnaires to those who provided consent at four time points: 3 months (T1), 6 months (T2), 12 months (T3), and 24 months (T4) post-diagnosis. Participants had the possibility to complete the questionnaire online (Qualtrics™, Provo, Utah, US). Summary of the tools and time point of measurement can be found in the Appendix (Appendix Table S1).

Follow-up reminders were sent after four weeks and phone calls were conducted if necessary. To support cohort retention, we sent greeting cards to the grandparents for major holidays and regular study updates. A clinical psychologist was available if needed. Enrollment was open from October 2020 to March 2023; follow-up was continued until December 2024. All paper responses were entered by a designated research staff member, and 20% of these entries were independently verified for accuracy. All datasets are stored on secure University servers with access restricted to authorized staff. Detailed study procedures can be found in a separate publication [19].

### Outcomes

#### Resilience

Resilience was assessed at T1-T4 using the Connor-Davidson Resilience Scale (CD-RISC 10), a 10-item self-report measure evaluating “positive adaptation in the face of stress or trauma” (Cronbach’s α = 0.93) [21, 22]. Items were rated on a 5-point Likert scale (0 = not true at all, 4 = true nearly all the time) and summed to create a resilience score. Missing responses were imputed using the mean of completed items when < 25% of items were missing. Summary of items can be found in the Appendix (Appendix Table S2).

#### Post-traumatic stress symptoms

Post-traumatic stress symptoms were measured at T3 and T4 using the Impact of Event Scale-Revised (IES-R), a 22-item self-report measure assessing subjective distress following traumatic events (Cronbach’s α = 0.91) [23]. For the current study, we adapted the IES-R, such that the traumatic event focused on the grandchild’s cancer diagnosis. The scale comprises three subscales: intrusion (8 items), avoidance (8 items), and hyperarousal (6 items). Items were rated on a 5-point Likert scale (0–4). Missing data (≤ 20% within a subscale) was imputed using the mean of completed subscale items. We only used the total score for this analysis ranging from 0 to 88, with higher scores indicating higher post-traumatic stress symptoms.

### Covariates and predictor variables

#### Sociodemographic and contextual factors (at T1)

We collected sociodemographic or contextual factors, namely, age, gender (male, female), language region (German, French/Italian), migration background (no migration background: those born in Switzerland and Swiss at birth, with migration background: all others), hospital proximity to the house of the grandparents (travel time measured in < 0.5 h, or ≥ 0.5 h), and number of grandchildren (≤ 2, > 2 grandchildren). All were collected at baseline (T1). Grandchild’s gender (male, female), grandchild’s age, cancer diagnosis (leukemia/lymphoma, other tumors), and therapy (chemotherapy, others) were obtained from medical records.

#### Internal (personal) resources

We also collected internal resources through the questionnaire, namely, household income satisfaction (“exceeds needs”;“meets needs”), education (compulsory school/vocational training, upper secondary/university), employment (unemployed/retired, employed), information received (medical information), general health, and health literacy.

Information needs and preferences were assessed at T1-T4 using a validated scale adapted from the Grandparents Information Needs Questionnaire (Cronbach’s α = 0.76) [24]. The instrument evaluated information needs across multiple domains: 9 medical items (cancer type, treatment, treatment goals, side-effects, disease progression, relapse, survival chance, late effects, palliative care) and 5 psychosocial items (communication with family, support for parents, support for grandchildren, own support, peer support). Participants indicated if they had received information (yes/no) for each domain. We calculated a score by counting the number of domains in which the participant received information.

General well-being was assessed using the first item of the Short Form-36 (SF-36) [25]: “In general, would you say your health is?” rated on a 5-point Likert scale (1 = poor, 5 = excellent).

Health literacy was measured at T3 using the European Health Literacy Survey Questionnaire (HLS-EU-Q12, Cronbach’s α = 0.84) [26] consisting of 12 items and assessing perceived difficulty in accessing, understanding, evaluating, and applying health information across healthcare, disease prevention, and health promotion domains. Items were rated on a 4-point Likert scale (1 = very easy, 4 = very difficult) and reverse-coded so higher scores indicated higher health literacy (sum score range: 12–48). Missing values were imputed using the median of the available responses when > 80% of items were completed.

#### External (social) resources

We collected external resources, namely, partnership status (no partner, in partnership), partnership quality, and social support.

Partnership quality was assessed using the Relationship-specific Attachment Scale for Adults (Beziehungsspezifische Bindungsskalen fur Erwachsene, Cronbach’s α = 0.78) [27], comprising 14 items rated on a 5-point Likert scale (1 = completely disagree, 5 = completely agree). Two subscales measured attachment security (6 items) and perceived available support (8 items). Negatively phrased items were reverse-coded. Subscale scores were summarized using means and standard deviations.

Perceived social support was measured at T1 using the Multidimensional Scale of Perceived Social Support (MSPSS, Cronbach’s α = 0.95) [28], a 12-item self-report measure assessing support from three sources (three subscales), namely, family, friends, and significant others (4 items each). Items were rated on a 7-point Likert scale (1 = very strongly disagree, 7 = very strongly agree). Missing data (if ≤ 1 item per subscale missing) was imputed using the mean of the completed items within the respective subscale. We summarized the response through sum score of all the items. Mean scores were calculated by averaging responses within each subscale.

### Data analysis

A framework for the analysis can also be found in the appendix (Appendix Figure S1). Stata 19.5 (StataCorp, Texas, US) was used for the analysis.

For aim 1, we used group-based trajectory modeling (GBTM) to identify trajectories of resilience. GBTM uses finite mixture models with censored normal distributions, treating the resilience sum score as a continuous variable. Trajectories were modeled using linear, quadratic, and cubic polynomial functions of time. Parameters were estimated using maximum likelihood estimation. Model fit was evaluated using AIC, BIC, and entropy. For participants with at most one missing response on the resilience scale, last observation carried forward imputation was applied. Analyses were conducted using the traj package [29]. We also explored the trajectories of grandparents’ resilience per patient (family) qualitatively.

For aim 2, we examined the associations between resilience trajectory group membership (identified in aim 1) and post-traumatic stress symptoms using Student’s t-test and linear mixed models (LMMs). For LMM, the IES-R sum score at T3 and T4 (continuous outcome) served as the dependent variable, and resilience trajectory group (categorical) as the independent variable. A random-intercept model was specified to account for repeated measures, with time included as a fixed effect. Models adjusted for age and sex were also fitted.

For aim 3, we assessed associations of internal and external resources with resilience using linear mixed-effects models, with the resilience sum score as the continuous outcome. Univariable linear mixed-effects models were fitted for each predictor. Fixed effects included time points (categorical, T1-T4), sociodemographic and contextual factors, internal resources, and external resources. A random-intercept model was used to account for repeated measures nested within participants.

### Ethical considerations

The study was approved by the Ethics Committee of Northwest and Central Switzerland (EKNZ 2020-01409, 26 August 2020). The study was done according to the Declaration of Helsinki and relevant national regulations. All participants provided written informed consent.

## Results

**Fig. 1:**
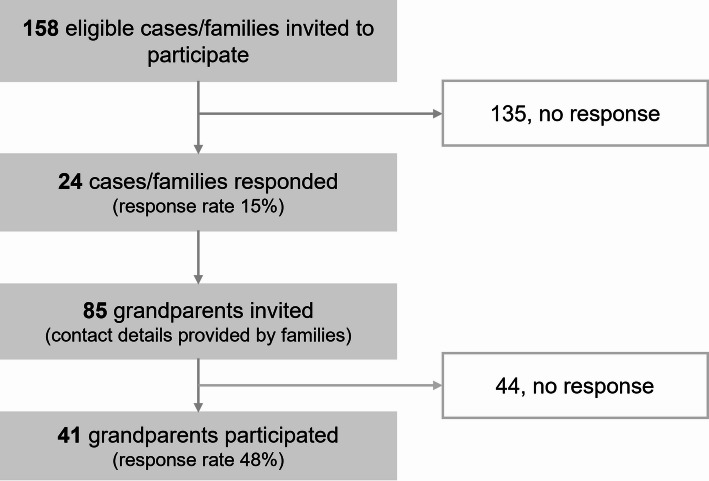
Flowchart of study participants

Of 85 grandparents invited to participate, 41 grandparents (25 grandmothers (61%); mean age: 67.6 years, range 55–80) of 20 grandchildren with cancer participated in the study (Table 1; Fig. 1). Total follow-up time has a mean of 21.4 months (SD 1.6) ranging from 18.2 to 24.5 months. Most participants were unemployed or retired (*n* = 23; 59%), and nearly all were in a partnership (*n* = 35, 90%). The grandchildren were predominantly female (*n* = 12, 63%) with a mean age of 6.0 years at diagnosis. Leukemia was the most frequent diagnosis (*n* = 10, 53%).

**Table 1:** Characteristics of participating grandparents (*n* = 41) and their grandchildren with cancer (*n* = 20)

Sociodemographic characteristics and contextual factors, *n* = 41		
Age, years		
Mean (SD)	67.6 (6.3)	
Range	55–80	
Sex		
Male	16	39%
Female	25	61%
Language region		
German	31	76%
French/Italian	10	24%
Migration background^1^		
No	34	87%
Yes	5	13%
Hospital proximity		
<0.5 h	10	27%
≥0.5 h	27	73%
Grandparent kinship		
Maternal grandmother	14	34%
Maternal grandfather	12	29%
Paternal grandmother	11	27%
Paternal grandfather	4	10%
Number of grandchildren		
≤2	16	42%
> 2	22	58%
**Child- and cancer-related characteristics**, *n* = 20		
Grandchild’s sex		
Female	12	63%
Male	7	37%
Grandchild’s age at diagnosis, years		
mean (SD)	6.0 (4.6)	
range	0–15	
Cancer diagnosis		
Leukemia/lymphoma	10	53%
CNS/solid tumors/others	9	47%
Therapy		
Chemotherapy	6	32%
Combination/surgery/radiotherapy	13	68%
**Internal (personal) resources**		
Household income satisfaction		
“Exceeds needs”	25	74%
“Meets needs”	9	26%
Education		
Compulsory school/Vocational training	23	60%
Upper secondary education/University	15	40%
Employment		
Unemployed/retired	23	59%
Employed	16	41%
General health^2^	2.28 (0.72)	
Health literacy^3^	57.2 (34.3)	
Information received^4^	7.5 (4.9)	
Medical information	5.4 (3.3)	
Psychosocial information	2.1 (2.1)	
**External (social) resources**		
Partnership		
No	4	10%
Yes	35	90%
Partnership quality^5^		
Attachment security	2.7 (0.3)	
Perceived partner support	3.3 (0.4)	
Social support^6^		
Overall	5.9 (0.8)	
Family	5.9 (0.9)	
Significant others	6.0 (1.2)	
Friends	6.0 (1.0)	

SD: Standard deviation; CNS: Central nervous system

^1^ Migration background are classified into none or Swiss: born in Switzerland and not naturalized or Swiss at birth. Otherwise, respondent is classified as someone with migration background

^2^ General health was assessed using the first question of Short Form-36 (SF-36) with (1 = excellent to 5 = poor). Expressed in mean and SD

^3^ Health literacy measured using European Health Literacy Survey Questionnaire (HLS-EU-Q12), expressed as percentage of ease of getting information for each domain (health care, disease prevention, and health promotion). Expressed in mean and SD

^4^ Information received measured using Grandparents Information Needs Questionnaire. It contains 14 items in total − 9 items medical information (cancer type, treatment, treatment goals, side-effects, disease progression, relapse, survival chance, late effects, palliative care) and 5 psychosocial information (communication with family, support for parents, support for grandchild, own support, peer support) items. Expressed in mean and SD

^5^ Partnership quality measured using Relationship-specific Attachment Scale for Adults (Beziehungsspezifische Bindungsskalen fur Erwachsene) with a scale of 1–5, including subscales. Expressed in mean and SD

^6^ Multidimensional scale of perceived social support scale (MSPSS) assessed support from family, significant other, and friends (1 = strongly disagree to 7-strongly agree)

### Trajectories of resilience (aim 1)

**Fig. 2:**
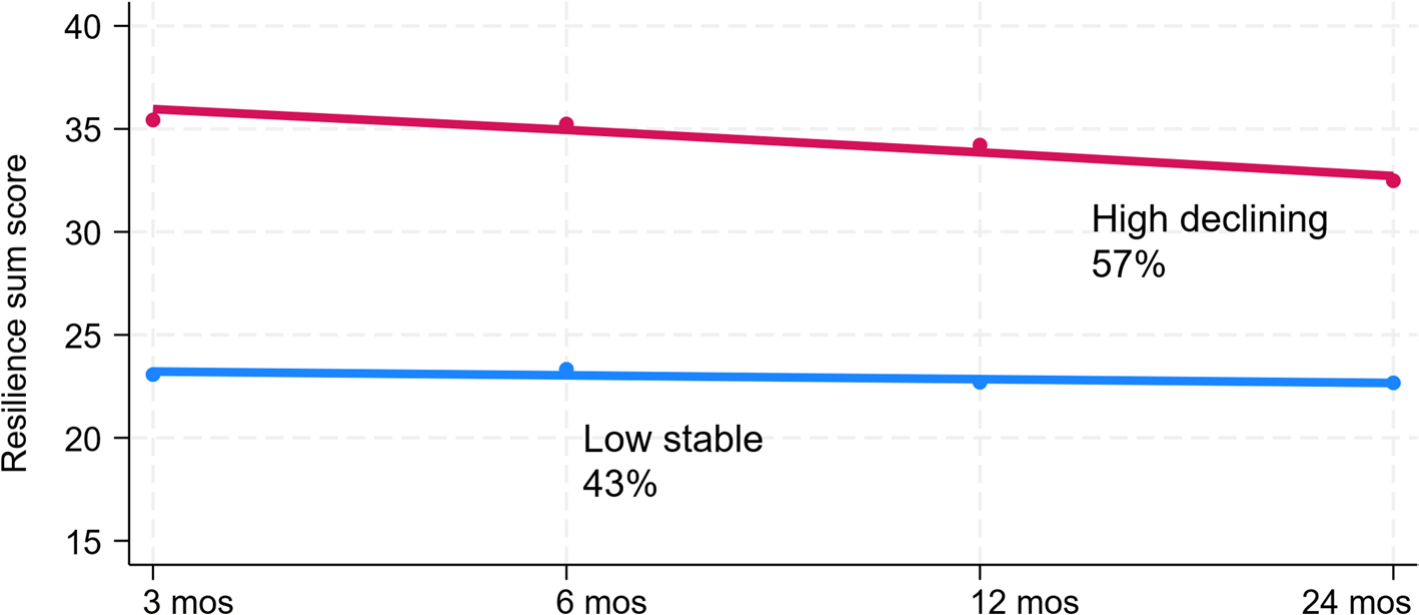
Resilience sum score in each time point for respective resilience trajectory *The y-axis was rescaled to allow closer examination of the declining trend at higher values. Lines (red and blue) depicts the trend in resilience sum scores across time. Linear equations can be found in the Appendix Table S3 (Model fit indices).

Detailed item responses of the participants on CD-RISC 10 can be found in the Appendix (Appendix Table S2). We identified two distinct trajectories of resilience among grandparents. The first trajectory, observed in 17 grandparents (43%), was characterized by consistently low resilience over time (low-stable trajectory). The second trajectory, observed in 23 grandparents (57%), started with higher resilience levels that declined over time (high-declining trajectory) (Fig. 2, Appendix Figure S2). Model fit indices and parameters for model selection can be found in the appendix (Appendix Table S3). Qualitative examination of resilience sum score over time appears to be similar within families (Appendix Figure S3).

### Association of resilience trajectories with post-traumatic stress symptoms (aim 2)

**Fig. 3:**
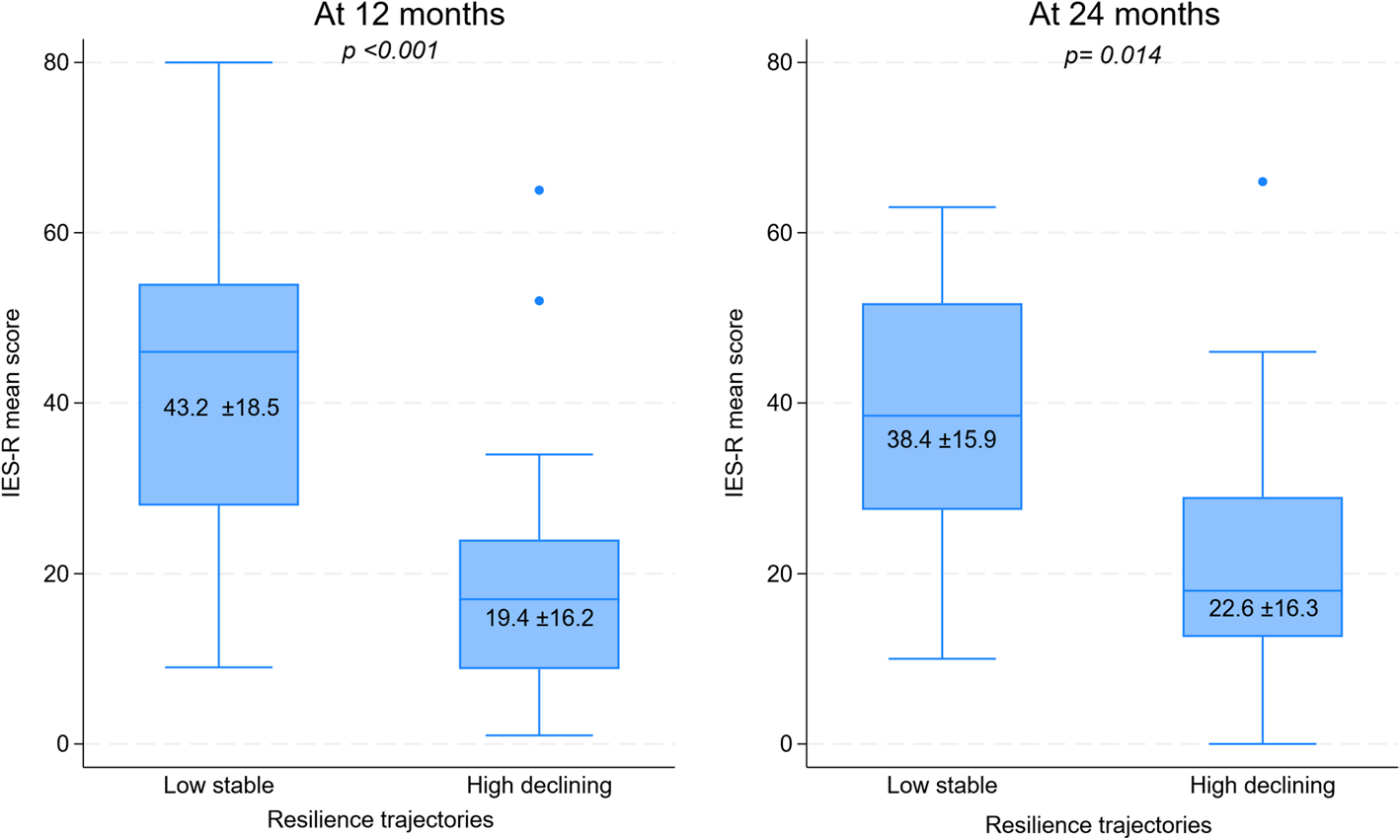
Post-traumatic stress (IES-R) in grandparents with Low-stable and High-decreasing level of resilience. Boxplot shows median, interquartile range, and range. Values in mean and standard deviation, p value from Student’s t-test.

The low-stable trajectory group had higher IES-R scores compared to the high-declining trajectory group at 12 months (mean = 43.2 SD = 18.5 vs. mean = 19.4 SD = 16.2, *p* < 0.001) and 24 months from the diagnosis (mean = 38.4 SD = 15.9 vs. mean = 22.6 SD = 16.3, p 0.014) (Fig. 3). Linear mixed models confirmed that those with low-stable resilience trajectory had higher post-traumatic stress symptoms compared to high-declining trajectory (β: −19.8, 90% CI −29.2, −10.4, *p* < 0.001; age-sex-adjusted model; β: −19.2, 90% CI −29.3, −9.1, *p* < 0.001; Appendix Table S4).

### Determinants of resilience (aim 3)

Higher health literacy (β: 0.3, 90% CI 0.20, 0.42, *p* < 0.001), information received in more domains (β: 1.53, 90% CI 1.27, 1.79, *p* < 0.001), and household income that “meets needs” (β: 7.56, 9% CI 1.86, 13.26, p 0.029) were all associated with higher resilience (Table 2). The finding on information received was consistent regardless of whether the information was medical or psychosocial. In contrast, none of the external resources were significantly associated with resilience in grandparents.

**Table 2:** Determinants of resilience (resilience sum score) among grandparents of grandchildren with cancer (*n* = 37; from univariable linear regression)

	Coefficient	90% CI	P value
**Sociodemographic characteristics**			
Age	−0.10	(−0.54, 0.35)	0.725
Sex			
Male -ref			
Female	−2.72	(−7.83, 2.40)	0.383
Language region			
German -ref			
Non-German	3.13	(−2.68, 8.94)	0.376
Migration background			
None-ref			
With migration background	2.69	(−5.18,10.57)	0.574
Hospital proximity			
<0.5 hour -ref			
>/= 0.5 hour	−3.75	(−9.66, 2.16)	0.297
Kinship			
Maternal grandmother -ref			
Maternal grandfather	1.80	(−5.66, 9.28)	0.635
Paternal grandmother	−0.41	(−8.06, 7.23)	0.916
Paternal grandfather	4.70	(−6.06, 15.47)	0.391
Number of grandchildren			
1 or 2 grandchildren -ref			
>2 grandchildren	−0.98	(−6.45, 4.49)	0.767
Grandchild’s sex			
Male-ref			
Female	−2.68	(−7.96, 2.60)	0.404
Grandchild’s age at diagnosis	−0.29	(−0.88, 0.29)	0.410
Diagnosis			
Leukemia/lymphoma -ref			
Others	0.65	(−4.61, 5.91)	0.840
Therapy			
Chemotherapy – ref			
Others	−0.07	(−5.29, 5.15)	0.983
**Internal (personal) resources**			
Income satisfaction			
“Exceeds needs” -ref			
“Meets needs”	7.56*	(1.86,13.26)	0.029
Education			
Low -ref			
High	4.18	(−1.16, 9.52)	0.198
Employment			
Unemployed/retired - ref			
Employed	0.22	(−5.15, 5.60)	0.945
General health	1.55	(−2.13, 5.23)	0.488
Health literacy	0.31*	(0.20, 0.42)	<0.001
Information received	1.53*	(1.27, 1.79)	<0.001
Medical information	2.32*	(1.97, 2.67)	<0.001
Psychosocial information	2.33*	(1.56, 3.09)	<0.001
**External (social) resources**			
Partnership			
No -ref			
Yes	−0.53	(−4.89, 3.82)	0.840
Partnership quality			
Attachment security	1.86	(−5.51, 9.23)	0.678
Perceived partner support	0.10	(−8.24, 8.43)	0.985
Social support			
Overall	0.14	(−2.70, 2.98)	0.936
Family	−0.86	(−3.86, 2.14)	0.638
Significant other	0.78	(−1.48, 3.04)	0.568
Friends	−0.04	(−2.73, 2.65)	0.980

CI: Confidence interval; SD: Standard deviation; CNS: Central nervous system; ref: Reference group

^1^ Migration background are classified into none or Swiss: born in Switzerland and not naturalized or Swiss at birth. Otherwise, respondent is classified as someone with migration background

^2^ General health was assessed using the first question of Short Form-36 (SF-36) with (1 = excellent to 5 = poor)

^3^ Health literacy measured using European Health Literacy Survey Questionnaire (HLS-EU-Q12), expressed as percentage of ease of getting information for each domain (health care, disease prevention, and health promotion)

^4^ Information received measured using Grandparents Information Needs Questionnaire. It contains 14 items in total, 9 items medical information (cancer type, treatment, treatment goals, side-effects, disease progression, relapse, survival chance, late effects, palliative care) and 5 psychosocial information (communication with family, support for parents, support for grandchild, own support, peer support) items

^5^ Partnership quality measured using Relationship-specific Attachment Scale for Adults (Beziehungsspezifische Bindungsskalen fur Erwachsene)with a scale of 1–5, including subscales

^6^ Multidimensional scale of perceived social support scale (MSPSS) assessed support from family, significant other, and friends (1 = strongly disagree to 7-strongly agree)

*p values < 0.05

## Discussion

Resilience plays an important role in shaping an individual’s response to traumatic stress, such as a grandchild’s cancer diagnosis. In our study, we identified two distinct resilience trajectories among grandparents of children with cancer: one with consistently low levels (low-stable) and another with initially high resilience that declined over time (high-declining). Grandparents in the low-stable group may have had higher levels of post-traumatic stress symptoms, suggesting that this subgroup is potentially vulnerable to long-term psychological distress. Income satisfaction, health literacy, and information received are internal resources that were associated with higher resilience in grandparents. Our findings provide novel insights into the psychological adaptation processes of grandparents during their grandchild’s cancer trajectory and highlight potential targets for intervention.

The high-declining resilience trajectory may reflect two distinct mechanisms. First, the initially elevated resilience may represent an adaptive stress response characterized by rapid mobilization of psychological resources during the acute crisis period compatible with the “shift-and-persist” model [30, 31]. This model shows that adaptation to stress through techniques like managing one’s emotions (shifting) and endure challenges by staying optimistic and finding purpose (persisting) could be a protective coping mechanism. This could be reflected as high resilience in our study [30, 31]. This initial surge in resilience may enable grandparents to maintain emotional stability, provide instrumental support to the family, and contribute to family cohesion during the most uncertain phases of diagnosis and treatment initiation. As treatment progresses and uncertainty possibly diminishes, resilience levels may naturally decline and return to pre-crisis baseline levels eventually, reflecting successful adaptation rather than psychological deterioration. This interpretation is supported by the concurrent decline in post-traumatic stress symptoms observed at T4, suggesting resolution of acute stress responses. Second, the resilience decline may reflect the cumulative burden of prolonged stress and emerging challenges during later treatment phases. Continuous or long-term exposure to stress may result in a decline in resilience, consistent with the stress-resilience theory [32–34]. Treatment completion, while symbolizing medical success, paradoxically removes the structured medical environment that provided clear objectives and frequent professional medical and institutional support. Our previous studies indicate that families continue to worry about recurrence, late effects, and the long-term psychosocial impact [35, 36]. Grandparents may also face new challenges in the post-treatment phase, such as navigating complex family readjustment processes related to the recovering child’s behavioral and developmental changes, difficulties in sibling adjustment, and intergenerational financial strain accumulated during treatment - all of which may contribute to decreasing resilience.

The low-stable resilience trajectory represents a particularly vulnerable subpopulation. Grandparents exhibiting this resilience trajectory had elevated levels of post-traumatic stress throughout the study period, potentially indicating inadequate stress processing and difficulty adapting to the cancer experience [3]. This pattern aligns with the view that resilience resembles a personality trait and represents a relatively stable construct [37]. Grandparents in this trajectory may have fewer coping strategies or pre-existing psychological vulnerabilities [37, 38]. Previous studies also found low resilience to be associated with poorer mental health outcomes, including depression, anxiety, adjustment disorders, and post-traumatic stress disorder [37]. Interventions improving resilience may help prevent physical and mental health disorders [39].

We found several factors and resources to be associated with resilience. Grandparents who had received more information about both psychosocial and medical topics showed higher resilience. This is in line with previous studies that demonstrated the importance of information delivery to grandparents of children with cancer [24, 40]. The mechanism underlying this association may involve enhanced cognitive control and reduced uncertainty through improved understanding of disease processes, treatment protocols, and expected outcomes. Access to comprehensible, relevant information enables grandparents to construct coherent mental models of the experience, facilitating predictive coping and proactive problem-solving strategies [41, 42]. This cognitive clarity enhances self-efficacy beliefs, promoting confidence in their ability to navigate complex medical environments and providing meaningful family support [41, 42]. Furthermore, knowledge acquisition regarding available psychosocial resources, communication strategies, and other families’ experiences contributes to an expanded coping repertoire and reduced feelings of isolation [10, 24, 43]. The information-resilience association may be bidirectional, as higher resilience may also motivate active information-seeking behavior, creating positive feedback loops that sustain adaptive functioning throughout the treatment trajectory.

We also found that grandparents reporting income that “meets needs” showed higher resilience than those reporting income that “exceeds needs.” This finding is counterintuitive, as most research suggests that greater financial resources support resilience [44]. Our measure was based on self-reported income satisfaction (“how well do you get al.ong with your household income”). Higher resilience among those who reported “meets needs” may reflect positivity and self-efficacy, which are part of the resilience construct [45]. However, it should be noted that those who report “meets needs” should be interpreted with caution due to limited statistical power.

The literature remains inconclusive regarding other determinants of resilience in older adults [41, 42, 46]. In our study, we observed no associations between social support, partnership quality, and resilience. Given the limited sample size, this null finding may reflect insufficient statistical power, precluding definitive interpretation. However, if true, it could indicate that resilience in grandparents is less influenced by external resources than previously assumed. Older adults typically exhibit higher baseline resilience due to accumulated life experience, repeated adversity, and long-term shaped coping strategies [47]. Resilience in later life is largely shaped by stable traits and coping patterns, consistent with the developmental timing hypothesis [48, 49]. Generational differences in help-seeking and social networks may further reduce the impact of current external resources, suggesting other unmeasured factors are more relevant in resilience building for this population.

## Strengths and limitations

To our knowledge, this study represents the first longitudinal examination of resilience among grandparents of children with cancer. The trajectory analysis approach, a perspective not previously explored in the literature, provides crucial insights into resilience evolution during their grandchild’s cancer journey. Identifying trajectories helped identify groups vulnerable for higher levels of post-traumatic stress symptoms. The multicenter design enhances representativeness and generalizability within the national context, while the sample size remains substantial given the rare events and recruitment challenges inherent in this population.

Several methodological limitations need to be highlighted to provide context in our findings. Selection bias represents a significant concern, as enrollment occurred through parental recruitment, potentially creating systematic exclusion of grandparents with strained family relationships, geographic barriers, or limited involvement in the child’s care. This recruitment strategy may have favored grandparents with stronger emotional investment, better family communication patterns, and greater social support networks, resulting in systematically higher resilience than would be observed in the broader grandparent population. The self-selection nature of participation further reinforces this bias, as grandparents willing to engage in our cohort study may possess inherently different psychological characteristics, including higher baseline resilience, better health status, or greater comfort with emotional disclosure. Thus, resilience estimates for our study may be higher than their true value. Measurement bias is an additional concern. CD-RISC 10 lacks established validation in the elderly Swiss population, raising questions about construct validity, cultural appropriateness, and normative interpretation of scores in the aging population [21]. Age-related cognitive changes, differential item interpretation, and cohort-specific understanding of resilience concepts [47] may influence response patterns in ways not captured by existing psychometric evaluations. Another limitation is the lack of detailed data on the grandchild’s clinical course, due to privacy restrictions. While resilience is a construct that is relatively stable, the clinical course may still affect post-traumatic stress and that those with low-stable resilience trajectory would have had higher stress from the experience. Religious and spiritual coping may also contribute to resilience by providing meaning, a sense of control, and interpretative frameworks for adverse life events [50, 51]. Future studies should examine their association with resilience outcomes. Finally, limited statistical power precluded some analytical approaches. We were unable to conduct complex multivariable regression analyses that could account for interactions among sociodemographic and contextual factors and resources. Trajectory analysis with only two identified groups may oversimplify the heterogeneity of resilience patterns, potentially masking additional meaningful subgroups or transition points.

## Implications and future research

Our findings reveal the impact of a diagnosis of childhood cancer on grandparents’ resilience. This underscores the importance of holistic care that includes not only the immediate family, but also grandparents and extended family members who might provide support. To help reduce grandparents’ anxiety and stress related to the cancer experience, a tailored information dissemination program is needed addressing grandparent-specific preferences and needs. Healthcare teams should encourage parents to be open to the inclusion of grandparents in family discussions, to ensure a comprehensive and family-centered care.

Future research should adopt family-centered approaches examining psychosocial outcomes across parents, siblings, and grandparents as integrated units. Additionally, investigation of intrafamilial dynamics and family resilience processes, currently focused on nuclear families, should expand to include grandparent contributions. Larger cohort studies are needed to explore complex associations and interactions and to identify additional factors influencing resilience in this understudied population.

## Conclusion

We identified two distinct resilience trajectories among grandparents following their grandchild’s cancer diagnosis: one characterized by initially high resilience that gradually declined, and another marked by persistently low resilience. Given the limited sample size, these findings should be interpreted with caution. The group with low resilience showed higher levels of post-traumatic stress symptoms, suggesting a potential need for targeted attention. Tailored, cancer-specific information delivery tailored to grandparents’ needs may enhance resilience and help prevent adverse psychological outcomes.

## Supplementary Information

Supplementary Material 1.

## Data Availability

Data used in the study is available to interested parties upon reasonable request from the corresponding author.
