# Supplementary material for: Resilience and post-traumatic stress symptoms in grandparents following their grandchild’s cancer diagnosis from a multicenter cohort study in Switzerland (The GROKids project)
Source: Discov Oncol. 2026 Feb 1;17:381. doi: 10.1007/s12672-026-04490-7 (PMC12953793; doi:10.1007/s12672-026-04490-7)
Supplement: Supplementary file 1 — Supplementary Material 1. [file 12672_2026_4490_MOESM1_ESM.docx]

Appendix (Supplementary Material)

Resilience and post-traumatic stress symptoms in grandparents following their grandchild’s cancer diagnosis from a multicenter cohort study in Switzerland (The GROkids Project)

Authors

Peter Francis Raguindin^1^, Anne Maas^1^, Anica Ilic^1,2^, Cristina Priboi^1, 3,^, Katharina Roser^1^, Ahmed Farrag^4,5^, Freimut Schilling^4^, Ursula Tanriver^6^, Tamara Diesch-Furlanetto^6^, Katrin Scheinemann^1,7^, Gisela Michel^1^

Affiliations

^1^ Faculty of Health Sciences and Medicine, University of Lucerne, Lucerne, Switzerland

^2^ Institute of Basic Medical Sciences, Faculty of Medicine, University of Oslo, Oslo, Norway

^3^ Veterinary Public Health Institute, University of Bern, Bern, Switzerland

^4^ Division of Pediatric Hematology and Oncology, Department of Pediatrics,

Children’s Hospital of Central Switzerland, Lucerne, Switzerland

^5^ Pediatric Oncology Department, South Egypt Cancer Institute, Assiut University, Assiut, Egypt

^6^ Department of Pediatric Oncology and Hematology, University Children’s Hospital Basel, Basel, Switzerland

^7^ Division of Hematology-Oncology, Children’s Hospital of Eastern Switzerland, St Gallen, Switzerland

Corresponding author

Prof. Dr. Gisela Michel

Faculty of Health Sciences and Medicine

University of Lucerne

Alpenquai 4, 6005 Lucerne

Email: gisela.michel@unilu.ch

Keywords/MeSH: resilience, post-traumatic stress, grandparents, family, cancer, childhood cancer

**Table S1.** Tools and time points at which they were measured

| Construct | Tool | T1 | T2 | T3 | T4 |
| --- | --- | --- | --- | --- | --- |
| Primary outcome |  |  |  |  |  |
| Resilience | Connor-Davidson Resilience Scale (CD-RISC 10) | X | X | X | X |
| Post-traumatic stress symptoms | Impact of Event Scale (IES-R) |  |  | X | X |
| Covariates |  |  |  |  |  |
| Sociodemographic information | Adaptation of the questionnaire from the Swiss Federal Statistical Office | X |  |  |  |
| Clinical information | Details on the cancer diagnosis and therapy |  |  |  | X |
| Information needs | Grandparents Information Needs Questionnaire | X |  |  |  |
| General wellbeing | First question of the Short-Form 36 | X |  |  |  |
| Health literacy | European Health Literacy Survey Questionnaire (HLS-EU-Q12) |  |  | X |  |
| Partnership quality | Relationship-specific Attachment Scale for Adults  (Beziehungsspezifische Bindungsskalen fur Erwachsene) | X |  |  |  |
| Perceived social support | Multidimensional Scale of Perceived Social Support (MSPSS) | X |  | X |  |

**Table S2.** Summary of item and item response by the participants in CD-RISC 10 (Resilience)

|  | T1  (3 months) | T2  (6 months) | T3  (12 months) | T4  (24 months) |
| --- | --- | --- | --- | --- |
| I am able to adapt when changes occur. (Positivity) | | | | |
| Not at all | - | - | 1 (2.9%) | - |
| Rarely true | 1 (2.6%) | 1 (3.0%) | - | - |
| Sometime true | 4 (10.3%) | 3 (9.1%) | 4 (11.8%) | 8 (26.7%) |
| Often true | 11 (28.2%) | 6 (18.2%) | 8 (23.5%) | 4 (13.3%) |
| Always true | 23 (59.0%) | 23 (69.7%) | 21 (61.8%) | 18 (60.0%) |
|  |  |  |  |  |
| I can deal with whatever comes my way. (Positivity) | | | | |
| Not at all | 1 (2.6%) | - | 1 (2.9%) | - |
| Rarely true | 2 (5.1%) | 1 (3.0%) | 1 (2.9%) | - |
| Sometime true | 9 (23.1%) | 9 (27.3%) | 6 (17.6%) | 10 (33.3%) |
| Often true | 12 (30.8%) | 15 (45.5%) | 16 (47.1%) | 14 (46.7%) |
| Always true | 15 (38.5%) | 8 (24.2%) | 10 (29.4%) | 6 (20.0%) |
|  |  |  |  |  |
| I try to see the humorous side of things when I am faced with problems. (Resistance) | | | | |
| Not at all | 3 (7.7%) | 3 (9.1%) | 3 (9.1%) | - |
| Rarely true | 4 (10.3%) |  | 6 (18.2%) | 3 (10.0%) |
| Sometime true | 13 (33.3%) | 12 (36.4%) | 11 (33.3%) | 11 (36.7%) |
| Often true | 10 (25.6%) | 12 (36.4%) | 9 (27.3%) | 9 (30.0%) |
| Always true | 9 (23.1%) | 6 (18.2%) | 4 (12.1%) | 7 (23.3%) |
|  |  |  |  |  |
| Having to cope with stress can make me stronger. (Resistance) | | | | |
| Not at all | 2 (5.1%) | - | 2 (6.1%) | 2 (6.7%) |
| Rarely true | 3 (7.7%) | 5 (15.2%) | 3 (9.1%) | 3 (10.0%) |
| Sometime true | 8 (20.5%) | 8 (24.2%) | 10 (30.3%) | 6 (20.0%) |
| Often true | 18 (46.2%) | 14 (42.4%) | 9 (27.3%) | 15 (50.0%) |
| Always true | 8 (20.5%) | 6 (18.2%) | 9 (27.3%) | 4 (13.3%) |
|  |  |  |  |  |
| I tend to bounce back after illness, injury or other hardships. (Positivity) | | | | |
| Not at all | 1 (2.6%) | - | 1 (2.9%) | - |
| Rarely true | 1 (2.6%) | 1 (3.0%) | 1 (2.9%) | 1 (3.3%) |
| Sometime true | - | 1 (3.0%) | 3 (8.8%) | 5 (16.7%) |
| Often true | 14 (35.9%) | 12 (36.4%) | 10 (29.4%) | 13 (43.3%) |
| Always true | 23 (59.0%) | 19 (57.6%) | 19 (55.9%) | 11 (36.7%) |
|  |  |  |  |  |
| I believe I can achieve my goals, even if there are obstacles. (Competence) | | | | |
| Not at all | - | - | 1 (2.9%) | - |
| Rarely true | 3 (7.9%) | - | 1 (2.9%) | 1 (3.3%) |
| Sometime true | 9 (23.7%) | 8 (24.2%) | 8 (23.5%) | 8 (26.7%) |
| Often true | 12 (31.6%) | 9 (27.3%) | 11 (32.4%) | 11 (36.7%) |
| Always true | 14 (36.8%) | 16 (48.5%) | 13 (38.2%) | 10 (33.3%) |
|  |  |  |  |  |
| Under pressure, I stay focused and think clearly. (Resistance) | | | | |
| Rarely true | 6 (15.4%) | 3 (9.1%) | 3 (9.1%) | 3 (10.0%) |
| Sometime true | 5 (12.8%) | 4 (12.1%) | 5 (15.2%) | 5 (16.7%) |
| Often true | 19 (48.7%) | 13 (39.4%) | 14 (42.4%) | 14 (46.7%) |
| Always true | 9 (23.1%) | 13 (39.4%) | 11 (33.3%) | 8 (26.7%) |
|  |  |  |  |  |

| Table S2. continued |  |  |  |  |
| --- | --- | --- | --- | --- |
|  | T1  (3 months) | T2  (6 months) | T3  (12 months) | T4  (24 months) |
| I am not easily discouraged by failure. (Competence) | | | | |
| Not at all | 1 (2.6%) | 2 (6.1%) | 3 (8.8%) | 1 (3.3%) |
| Rarely true | 5 (12.8%) | 3 (9.1%) | 3 (8.8%) | 4 (13.3%) |
| Sometime true | 9 (23.1%) | 8 (24.2%) | 6 (17.6%) | 9 (30.0%) |
| Often true | 15 (38.5%) | 11 (33.3%) | 11 (32.4%) | 8 (26.7%) |
| Always true | 9 (23.1%) | 9 (27.3%) | 11 (32.4%) | 8 (26.7%) |
|  |  |  |  |  |
| I think of myself as a strong person when dealing with life’s challenges and difficulties. (Competence) | | | | |
| Not at all | 1 (2.6%) | - | - | - |
| Rarely true | 1 (2.6%) | 2 (6.1%) | 2 (6.1%) | 1 (3.3%) |
| Sometime true | 6 (15.4%) | 4 (12.1%) | 4 (12.1%) | 8 (26.7%) |
| Often true | 16 (41.0%) | 9 (27.3%) | 9 (27.3%) | 10 (33.3%) |
| Always true | 15 (38.5%) | 18 (54.5%) | 18 (54.5%) | 11 (36.7%) |
|  |  |  |  |  |
| I am able to handle unpleasant or painful feelings like sadness, fear, and anger. (Resistance) | | | | |
| Not at all | - | 1 (3.0%) | 2 (5.9%) | - |
| Rarely true | 5 (12.8%) | 2 (6.1%) | 1 (2.9%) | 3 (10.0%) |
| Sometime true | 9 (23.1%) | 8 (24.2%) | 10 (29.4%) | 9 (30.0%) |
| Often true | 10 (25.6%) | 9 (27.3%) | 10 (29.4%) | 8 (26.7%) |
| Always true | 15 (38.5%) | 13 (39.4%) | 11 (32.4%) | 10 (33.3%) |

Briefly, the definitions of CD-RISC subscales (shown in parenthesis) are as follows:

1. Competence was defined as belief in one’s ability, having high standards, and tenacity.
2. Resistance corresponds to trust in one’s instincts, tolerance of negative affect, and strengthening effects of stress.
3. Positivity was a construct that relates to the positive acceptance of change, and secure relationships.


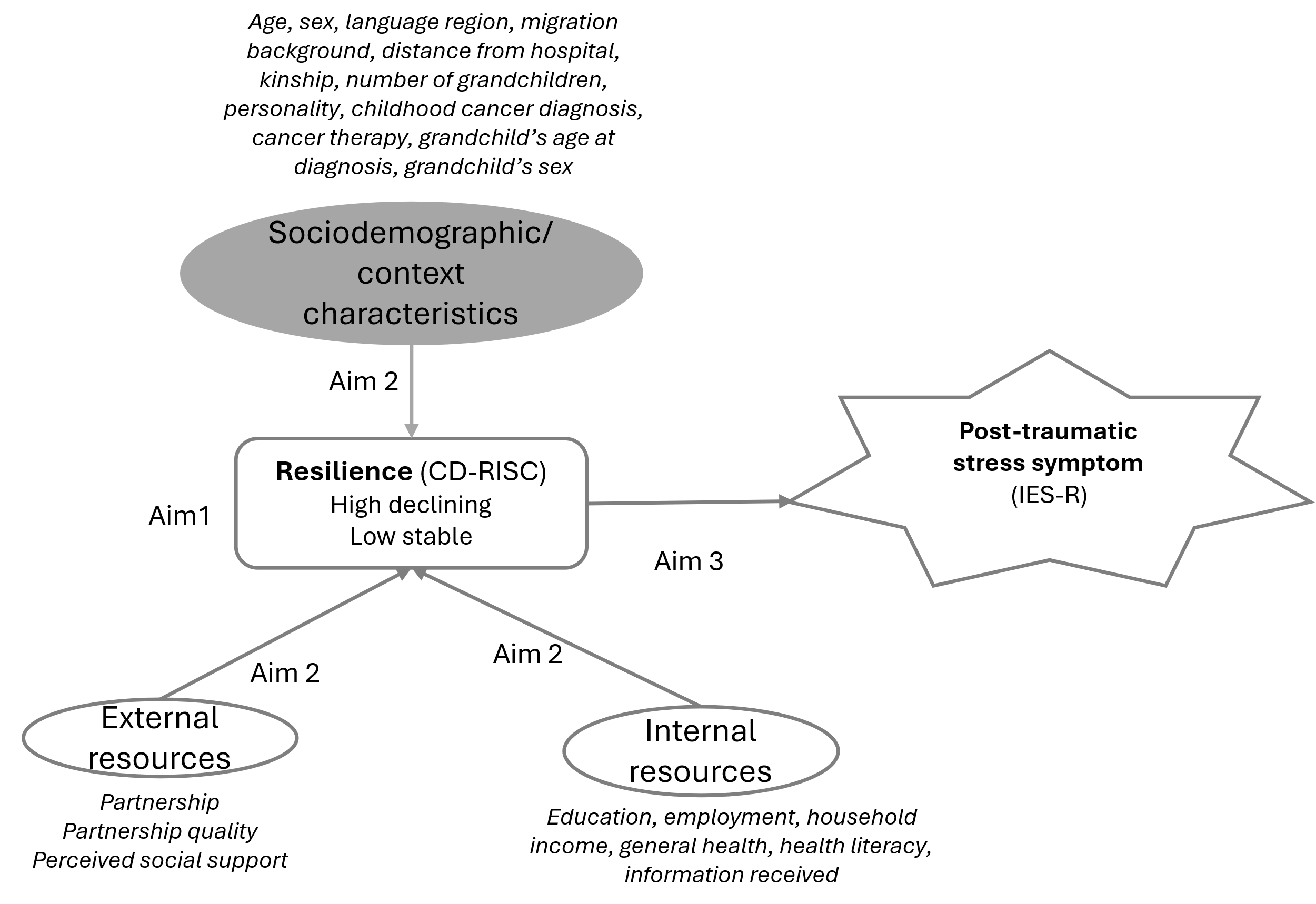


**Figure S1.** Analysis framework

**Table S3.** Model fit indices on the trajectories of resilience

| **TWO GROUP MODELS** | | | | | | |
| --- | --- | --- | --- | --- | --- | --- |
|  | Beta (SE) | Intercept | p value | AIC | BIC | Entropy |
| A. Linear - Linear |  |  |  |  |  |  |
| Group 1 - Linear | -0.02 (0.54) | 23.7 | 0.968 | -425.96 | -431.02 | 0.859 |
| Group 2 - Linear | -1.15 (0.46) | 37.3 | 0.013* |  |  |  |
|  |  |  |  |  |  |  |
| B. Linear-Quadratic |  |  |  |  |  |  |
| Group 1 - Linear | -0.01 (0.54) | **23.6** | **0.985** |  |  | 0.858 |
| Group 2 - Quadratic | -0.43 (0.51) | 35.0 | 0.394 | -426.95 | -432.86 |  |
|  |  |  |  |  |  |  |
| C. Quadratic - Linear |  |  |  |  |  |  |
| Group 1 - Quadratic | -0.07 (0.62) | 23.4 | 0.907 | -426.95 | -432.86 | 0.858 |
| Group 2 - Linear | -1.15 (0.46) | 37.3 | 0.014* |  |  |  |
|  |  |  |  |  |  |  |
| D. Quadratic-Quadratic |  |  |  |  |  |  |
| Group 1 - Quadratic | -0.05 (0.62) | 23.4 | 0.926 | -427.57 | -434.33 | 0.857 |
| Group 2 - Quadratic | -0.43 (0.51) | 35.1 | 0.397 |  |  |  |
|  |  |  |  |  |  |  |
| **THREE GROUP MODELS** | | | | | | |
|  | Beta (SE) | Intercept | p value | AIC | BIC | Entropy |
| A. 3 Linear |  |  |  |  |  |  |
| Group 1 - Linear | 0.25 (0.51) | 21.9 | 0.616 | -411.33 | -418.93 | 0.881 |
| Group 2 - Linear | -0.89 (0.46) | 33.6 | 0.054 |  |  |  |
| Group 3 - Linear | -2.60 (1.58) | 45.9 | 0.103 |  |  |  |
|  |  |  |  |  |  |  |
| B. 3 Quadratic |  |  |  |  |  |  |
| Group 1 - Quadratic | 0.001 (0.58) | 21.8 | 0.995 | -414.06 | -424.19 | 0.871 |
| Group 2 - Quadratic | -0.22 (0.48) | 32.3 | 0.640 |  |  |  |
| Group 3 - Quadratic | -0.44 (0.48) | 42.4 | 0.581 |  |  |  |
|  |  |  |  |  |  |  |
| C. 3 Cubic |  |  |  |  |  |  |
| Group 1 - Cubic | 0.04 (0.88) | 21.4 | 0.958 | -416.97 | -429.64 | 0.871 |
| Group 2 - Cubic | 0.05 (0.72) | 31.7 | 0.939 |  |  |  |
| Group 3 - Cubic | 0.45 (1.27) | 37.6 | 0.721 |  |  |  |
|  |  |  |  |  |  |  |
| D. Different shapes |  |  |  |  |  |  |
| Group 1 - Linear | 0.26 (0.51) | 21.8 | 0.612 | -413.06 | -422.35 | 0.871 |
| Group 2 - Quadratic | -0.22 (0.48) | 32.3 | 0.639 |  |  |  |
| Group 3 - Quadratic | -0.44 (0.80) | 37.6 | 0.580 |  |  |  |
|  |  |  |  |  |  |  |
| E. Different shapes |  |  |  |  |  |  |
| Group 1 - Linear | 0.26 | 21.8 | 0.616 | -413.98 | -424.11 | 0.871 |
| Group 2 - Quadratic | -0.22 | 32.3 | 0.634 |  |  |  |
| Group 3 - Cubic | 0.45 | 37.6 | 0.717 |  |  |  |
|  |  |  |  |  |  |  |

Abbreviations: AIC, Akaike Information Criterion, BIC, Bayesian Information Criterion.

Model in gray is the chosen model.


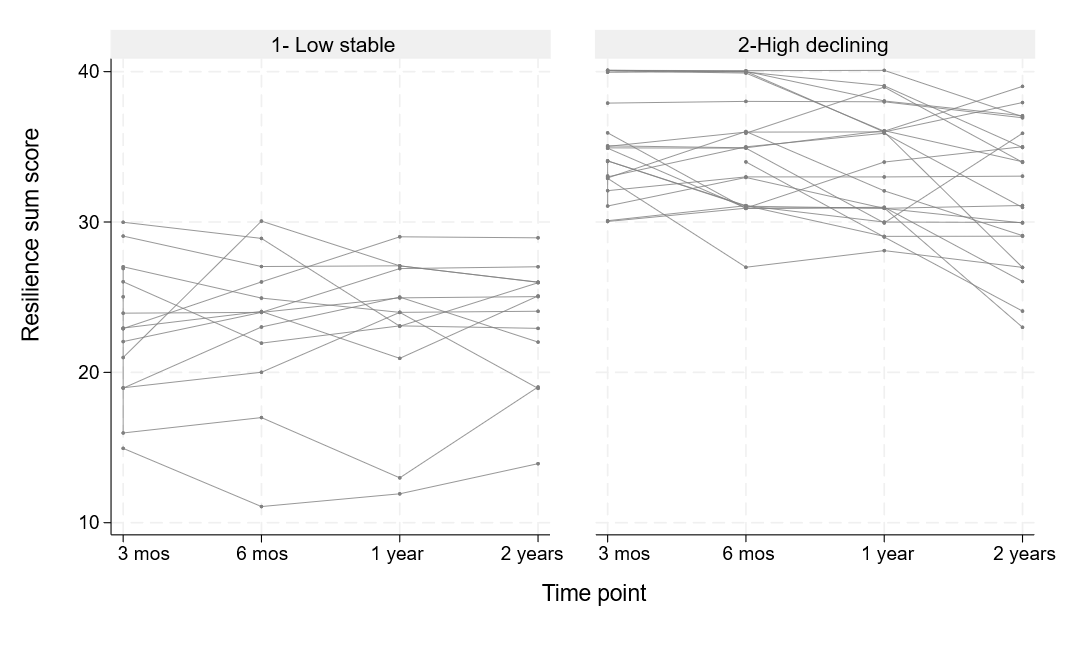


**Figure S2**. Trajectory plot of each participant divided into trajectory of resilience.

*Each line represents one participant with each point is a measure for each time point.


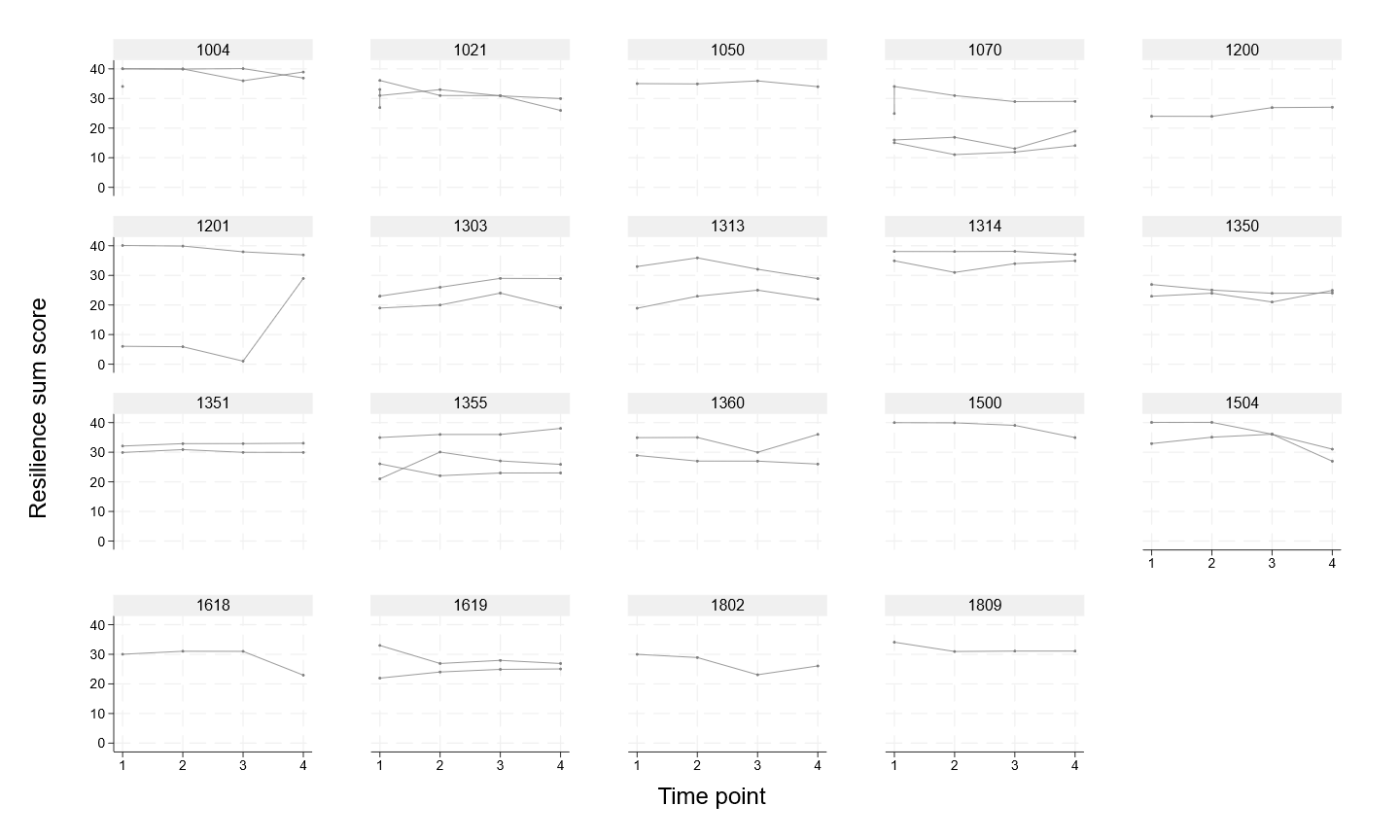


**Figure S3.** Trajectory plot of resilience sum score per family (patient).

*One grandparent (of one family) was removed from contributing only one time point

**Table S4.** Association of resilience trajectories (low-stable vs high-declining) and post-traumatic stress symptoms.*

|  | Crude | | | Adjusted^1^ | | |
| --- | --- | --- | --- | --- | --- | --- |
|  | beta | 90% CI | p-value | beta | 90% CI | p-value |
| IES-R mean score difference | -19.80 | -29.16, -10.43 | <0.001 | -19.19 | -29.26, -9.13 | <0.001 |
|  |  |  |  |  |  |  |
| Intercept | 40.79 | 33.65, 47.95 | <0.001 | 59.36 | 6.04, 112.6 | 0.029 |

*Regression using IES-R sum score as dependent variable and resilience trajectory group (Low-stable as reference) as independent variable. Linear mixed model fitted using individual as cluster and random-intercept model.

^1^Adjusted by age and sex.

Abbreviation: IES-R, Impact of Event Scale Revised
